# Immune Evasion by CMV and BK Virus: Lurking Viruses Waiting for the Right Moment to Deliver a Fatal Blow to Individuals With Compromised Immunity

**DOI:** 10.1155/jimr/2341054

**Published:** 2026-10-21

**Authors:** Luobin Robin Zheng, Maike Zheng, Yongqiang Wang, Shijun J. Zheng

**Affiliations:** ^1^ State Key Laboratory of Veterinary Public Health and Safety and Key Laboratory of Animal Epidemiology of the Ministry of Agriculture College of Veterinary Medicine at China Agricultural University Beijing 100193 China; ^2^ State Key Laboratory of Molecular Oncology National Cancer Center National Clinical Research Center for Cancer Cancer Hospital Chinese Academy of Medical Sciences and Peking Union Medical College Beijing 100021 China cacms.ac.cn

**Keywords:** BK virus (BKV), cytomegalovirus (CMV), immune evasion, immunocompromised host, immunotherapy

## Abstract

Human cytomegalovirus (CMV) and BK polyomavirus are archetypal latent DNA viruses that establish lifelong persistence and cause severe disease upon reactivation in immunocompromised hosts. Although they differ markedly in virion architecture, genome size, and coding capacity, both viruses converge on a shared biological principle: durable persistence is achieved through sustained immune invisibility rather than maximal replication. CMV is a large, enveloped double‐stranded DNA (dsDNA) virus whose multilayered structure supports the expression of numerous immunomodulatory proteins that disrupt antigen processing and presentation, modulate natural killer cell surveillance, and reshape cytokine and chemokine networks. In contrast, BK virus (BKV) is a small, nonenveloped dsDNA virus that achieves immune evasion through functional minimalism, restricting viral gene expression, limiting antigen availability, and attenuating innate immune sensing pathways. These distinct yet convergent immune evasion strategies complicate diagnosis, constrain the efficacy of antiviral therapies, and promote viral persistence in vulnerable patients. By integrating structural and biological features with mechanisms of immune escape, this review primarily focuses on how immune modulation blurs the classical distinction between latency and active infection, highlighting implications for clinical monitoring, therapy, and emerging immune‐guided strategies for the effective control of viral infection in immunocompromised individuals.

## 1. Introduction

Cytomegalovirus (CMV) and BK virus (BKV) are widespread DNA viruses that remain in the body after primary infection [1, 2]. In human with a normal immune system, both viruses stay at low levels of copies and rarely cause symptoms [1, 3]. When host immunity is supressed, CMV and BKV can be reactivated, leading to serious diseases [4, 5]. This often happens in patients who have received solid‐organ or stem‐cell transplants, undergone chemotherapy, or reached the late stage of HIV infection [3, 6]. Under these conditions, CMV reactivation may cause pneumonia, gastrointestinal injury, retinitis, or systemic inflammation [2, 3]. BKV reactivation is strongly linked to polyomavirus‐associated nephropathy in kidney‐transplant recipients and to hemorrhagic cystitis after stem‐cell transplantation [7]. These complications threaten transplant patient survival and their long‐term outcomes.

A major reason why CMV and BKV remain difficult to manage is their strong ability to escape immune control [1, 8]. Each virus uses a broad set of strategies to hide from host surveillance, suppress antiviral responses, and maintain long‐term persistence [1, 8]. They interfere with innate immune recognition, reduce antigen presentation, and restrict the activity of T cells and NK cells [8, 9]. As a consequence, both viruses can lurk in a latent state and re‐emerge when host immunity becomes insufficient.

This review aims to summarize the structural and genomic features of CMV and BKV, outline their major immune‐evasion strategies, and explain how these mechanisms contribute to clinical diseases in immunocompromised hosts. By examining two biologically distinct yet clinically parallel viruses, this article has provided a clear view of how viral latency, immune manipulation, and host vulnerability interact to produce high‐risk infections.

## 2. Structural and Genomic Features of CMV and BKV

### 2.1. CMV

CMV is a large, enveloped double‐stranded DNA (dsDNA) virus, belonging to the CMV genus of Beta‐herpesvirinae subfamily at Herpesviridae family [10]. As shown in Figure 1, the virion of CMV is typically 200–300 nm in diameter, exhibiting a multilayered architecture composed of four major components [10, 13]. At its core lies an icosahedral nucleocapsid enclosing a linear dsDNA genome of approximately 235 kb [10, 13]. This genome, one of the largest among human herpesviruses, contains more than 200 open reading frames (ORFs) encoding proteins that regulate distinct stages of the viral replication cycle [10, 14]. Surrounding the nucleocapsid is a dense tegument layer containing numerous viral and host‐derived proteins involved in immune modulation, replication, and virion assembly [10]. Among CMV structural components, tegument proteins are key early effectors that help both initiate infection and decrease host antiviral defenses [15]. In particular, pp71 (UL82) is released into the host cell upon entry and promotes immediate early gene expression by counteracting Daxx‐mediated intrinsic repression of the incoming viral genome [15, 16]. In parallel, the abundant tegument protein pp65 (UL83) dampens early innate antiviral responses, thereby creating a more permissive intracellular environment for viral replication [15]. The entire virion is enveloped by a host‐derived lipid membrane, which is embedded with viral glycoprotein complexes responsible for host‐cell attachment and membrane fusion [11, 13, 17].

**Figure 1:**
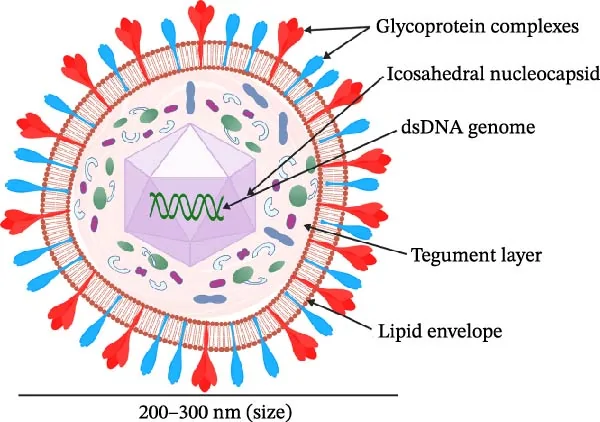
Schematic illustration of the CMV structure, consisting of the envelope, tegument, nucleocapsid, and dsDNA genome. The CMV genome has a unique organization consisting of long and short segments flanked by inverted repeats, enabling genome isomerization and contributing to viral adaptability [11]. This complex structural and genomic organization supports CMV’s capacity to establish lifelong latency and evade host immune surveillance [11, 12]. The underlying molecular mechanism needs further investigation. This diagram is adapted from previous publication [13]. Created with BioGDP.com.

### 2.2. BKV

BKV, a small nonenveloped dsDNA virus, belongs to the *Betapolyomavirus* genus of the Polyomaviridae family [18]. As shown in Figure 2, BKV has a diameter of approximately 40–45 nm and displays a simple icosahedral capsid structure composed of 72 pentameric capsomers of the major structural protein VP1, which encapsidate the circular dsDNA genome of about 5 kbp [19]. Minor structural proteins VP2 and VP3 are located beneath the capsid and play crucial roles in viral entry and genome delivery into the host nucleus [18, 20, 21].

**Figure 2:**
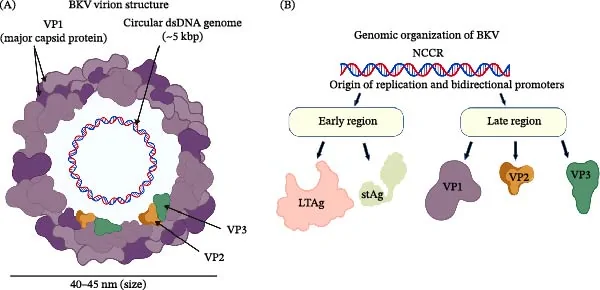
Structural and genomic organization of BKV. (A) Schematic illustration of BKV virion with the small, nonenveloped icosahedral capsid (40–45 nm in diameter) composed of 72 VP1 pentamers that encapsidate the circular double‐stranded DNA genome (~5 kb). Minor capsid proteins VP2 and VP3 are located beneath the VP1 shell and contribute to viral entry and genome delivery into the host nucleus. (B) Genome organization of BKV showing the noncoding control region (NCCR), which contains the origin of replication and bidirectional promoters regulating transcription of the early and late regions. The early region encodes the regulatory proteins large T antigen (LTAg) and small t antigen (stAg), whereas the late region encodes the structural proteins VP1, VP2 and VP3. Created with BioGDP.com.

The BKV genome is organized into three functional regions [22]. The early region, encoding regulatory proteins (large T antigen [LTAg] and small t antigen), is responsible for viral replication and cell cycle modulation [22–25]. The late region encoding the capsid proteins (VP1, VP2, and VP3) [22, 26] and the noncoding control region (NCCR), which contains the origin of replication and bidirectional promoters regulating gene expression [22, 27, 28]. This compact genomic organization allows efficient replication within host nuclei and contributes to viral persistence in renal epithelial cells [29]. The absence of a lipid envelope makes BKV highly stable in the extracellular environment [30, 31]. Such structural and genomic simplicity combined with its efficient replication strategy underlies BKV’s ability to establish latent infections and reactivate under immunosuppressive condition [1, 32].

### 2.3. Functional Implications of Structural Components

The structural elements of CMV and BKV directly shape their infection strategies and interactions with the host. For CMV, the lipid envelope and its glycoprotein complexes determine receptor binding and fusion pathways, enabling broad cell entry [33, 34]. The tegument delivers immune‐modulatory and replication‐initiating proteins immediately upon infection, giving CMV an advantage before viral gene expression begins [34]. Its large nucleocapsid and segmented genome organization support flexible gene regulation, latency, and rapid adaptation within host cells [33].

For BKV, the nonenveloped capsid provides environmental stability and facilitates transmission [35]. VP1 dictates receptor specificity, while exposure of VP2/VP3 during entry promotes membrane penetration and nuclear delivery of the circular genome [18]. The compact architecture with regulatory elements in the NCCR allows efficient replication and a switch between latency and reactivation under immunosuppressive condition [18, 36]. These two viruses achieve persistent infections through fundamentally different strategies. CMV evades the immune system by leveraging its multilayered complexity and BKV by capitalizing on a simpler yet highly stable capsid design.

The major structural and genomic differences between CMV and BKV are summarized in Table 1.

**Table 1:** Comparison of the structural and genomic characteristics of CMV and BKV.

Characteristic	CMV	BKV	Function	References
Virus family	Herpesviridae	Polyomaviridae	Represent distinct DNA virus families with different evolutionary backgrounds.	[10, 18]
Genome	Linear dsDNA (~235 kb)	Circular dsDNA (~5 kb)	Genome organization determines viral coding capacity and replication strategy.	[10, 13, 19]
Envelope	Present	Absent	The CMV envelope mediates host‐cell attachment and membrane fusion, whereas the nonenveloped BKV capsid enhances environmental stability.	[11, 13, 17, 30, 31]
Virion size	200–300 nm	40–45 nm	Reflects differences in virion complexity and structural organization.	[10, 13, 19]
Major structural proteins	Envelope glycoproteins, tegument proteins, and capsid proteins	VP1, VP2, VP3	Structural proteins mediate viral attachment, entry, genome delivery, and virion assembly.	[10, 11, 15, 17, 18, 20, 21, 26]
Genome organization	UL and US regions flanked by inverted repeats	Early region, late region, and noncoding control region (NCCR)	Different genome organizations regulate viral gene expression and replication.	[11, 12, 22, 27, 28]
Structural features	Multilayered virion containing envelope, tegument, and nucleocapsid	Compact icosahedral capsid lacking an envelope	CMV relies on structural complexity, whereas BKV relies on capsid stability for persistent infection.	[10, 13, 18, 19, 30, 31, 33–36]

## 3. Viral Attachment, Entry, and Cellular Tropism of CMV and BKV

Attachment and entry are critical determinants of viral host range, tissue tropism, and disease pathogenesis.

### 3.1. CMV: Glycoprotein‐Driven Entry and Broad Tropism

CMV entry is a multistep process coordinated by envelope glycoprotein complexes that determine receptor usage, mode of internalization, and cellular tropism [33]. Among these, the gH/gL/gO trimer and the gH/gL/UL128/UL130/UL131A pentamer are the principal entry complexes and play distinct roles in the infection of different cell types [33, 37]. Through differential use of these complexes, CMV is able to infect a wide range of cells, including fibroblasts, epithelial cells, endothelial cells, and cells of myeloid origin [17].

In fibroblasts, CMV entry is mediated primarily by the gH/gL/gO trimer and is strongly associated with engagement of platelet‐derived growth factor receptor alpha (PDGFRA), leading predominantly to fusion at the plasma membrane [17, 37, 38]. By contrast, infection of epithelial, endothelial, dendritic, or other myeloid cells depends mainly on the pentameric complex, with neuropilin‐2 (NRP2) identified as a major receptor facilitating entry into these cell types [39, 40]. In these settings, virions are commonly internalized through endocytic pathways, after which endosomal acidification promotes membrane fusion and release of the capsid into the cytoplasm [17]. The ability to utilize distinct glycoprotein complexes and entry routes enables CMV to establish infection across a broad range of tissues and cell populations [38] (Figure 3).

**Figure 3:**
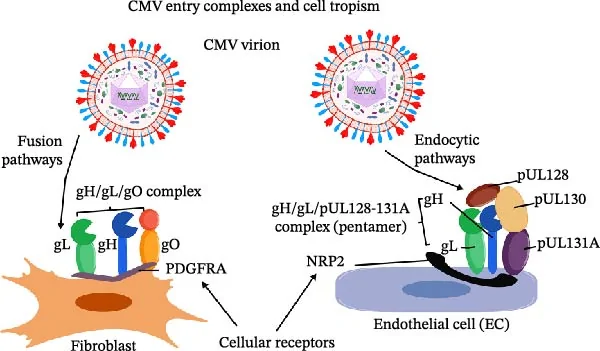
Cell type‐specific entry pathways of CMV. CMV employs two principal glycoprotein complexes for cell entry. In fibroblasts, the gH/gL/gO trimer engages PDGFRα and mediates fusion at the plasma membrane. In epithelial and endothelial cells, the gH/gL/UL128‐131 pentamer binds NRP2 and enters via endocytic pathways, with endosomal acidification triggering membrane fusion. Differential use of these complexes allows HCMV to infect diverse cell types. Created with BioGDP.com.

### 3.2. BKV: Glycan Receptor Engagement and Restricted Tropism

In contrast to CMV, BKV is a small, nonenveloped polyomavirus with a more restricted cellular tropism [41]. Initial attachment is mediated by the major capsid protein VP1, which binds specific sialylated glycosphingolipids on the host‐cell surface, particularly the gangliosides GD1b and GT1b [42, 43]. Recognition of terminal α2,8‐linked disialic acid motifs by VP1 is a key determinant of productive infection and contributes substantially to the selective tropism of BKV [43].

Following receptor binding, BKV is internalized mainly through caveolae‐dependent or lipid raft‐associated endocytosis [44, 45]. The virus is subsequently trafficked through intracellular vesicular compartments to the endoplasmic reticulum, where capsid rearrangement and partial disassembly occur [46]. These events facilitate membrane penetration and subsequent nuclear delivery of the viral genome, allowing the initiation of viral replication [46]. Because this entry pathway depends on both specific glycan receptors and compatible intracellular trafficking machinery [45], BKV infection is largely restricted to renal tubular epithelial cells and urothelial cells, consistent with its major clinical manifestations [46], including BKV‐associated nephropathy and hemorrhagic cystitis [5] (Figure 4).

**Figure 4:**
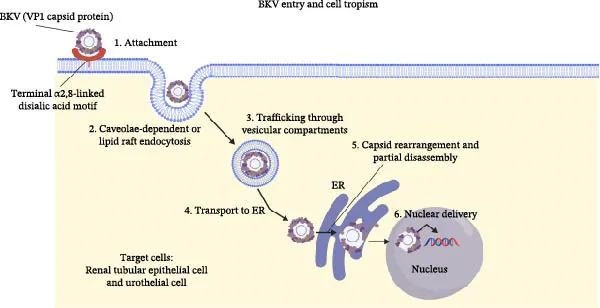
Schematic overview of BKV entry and restricted tropism. Following receptor engagement, BKV undergoes endocytic internalization, trafficking to the ER, capsid remodeling, and nuclear genome delivery. Infection is predominantly confined to renal tubular and urothelial cells. Created with BioGDP.com.

CMV achieves broad tropism via the differential use of trimeric and pentameric glycoprotein complexes engaging distinct receptors, whereas BKV employs VP1‐mediated glycan recognition and caveolae‐dependent entry, restricting infection to renal and urothelial cells.

## 4. Mechanisms of Immune Evasion by CMV

CMV employs coordinated strategies to evade both adaptive and innate immune surveillance, primarily by disrupting antigen presentation to CD8^+^ T cells and modulating NK cell recognition (Figure 5), thereby establishing a persistent infection.

**Figure 5:**
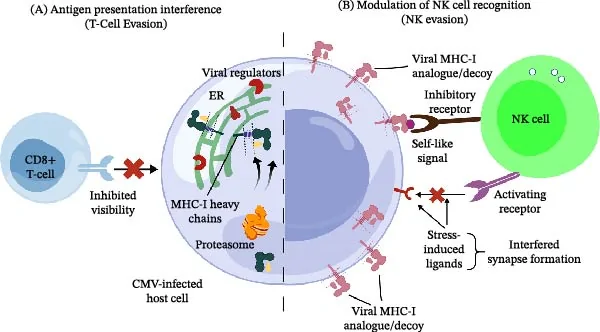
Cellular mechanisms of immune evasion by CMV. (A) CMV interferes with MHC‐I antigen processing and presentation by retaining, degrading, or misrouting MHC‐I molecules, thereby reducing CD8^+^ T‐cell recognition of infected cells. (B) To counterbalance MHC‐I loss‐induced NK cell activation, CMV expresses MHC‐I‐like decoy ligands and suppresses stress‐induced activating signals, thereby inhibiting NK cell cytotoxicity and immune synapse formation. Created with BioGDP.com.

### 4.1. Antigen Presentation Interference

CMV employs an extensive repertoire of viral proteins to prevent effective antigen display on host cells [47, 48]. Several CMV gene products block distinct steps along the MHC‐I pathway [49]. For example, certain viral regulators retain newly synthesized MHC‐I heavy chains in the endoplasmic reticulum, redirect them for proteasomal degradation, or inhibit peptide loading [50]. These viral regulators include US2, US3, US6, and US11, which target distinct steps in the MHC‐I antigen‐presentation pathway [51–53]. US2 and US11 promote degradation of MHC‐I heavy chains, US3 retains MHC‐I in the endoplasmic reticulum and,US6 inhibits TAP‐dependent peptide transport [51–53]. This process sharply reduces the quantity of peptide–MHC‐I complexes presented at the plasma membrane [49]. CMV secures a window of replication before adaptive immunity responds by inhibiting the visibility of infected cells to CD8^+^ cytotoxic T lymphocytes [54, 55].

### 4.2. Modulation of NK Cell Recognition

CMV simultaneously has more strategies to resist innate immune surveillance since the lack of MHC‐I caused NK‐cell‐mediated death [56]. The virus expresses several MHC‐I analog to decoy molecules that engage inhibitory NK‐cell receptors and recreate a “self‐like” signal despite profound disruption of genuine MHC‐I presentation [57, 58]. A well‐characterized example is UL18, a viral MHC‐ I homolog that engages the inhibitory receptor LIR‐1/ILT2 on NK cells and helps prevent NK‐cell‐mediated killing [57, 58]. Other CMV proteins downregulate stress‐induced ligands that normally activate NK cells, while additional viral factors interfere with NK cell receptor–ligand interactions that are required for immune synapse formation [59–61]. For example, UL16 retains MICB, ULBP1, and ULBP2, while UL142 reduces the surface expression of MICA, thereby limiting activation through NKG2D [62–64].

### 4.3. Cytokine and Chemokine Network Manipulation

The cytokine‐ and chemokine‐based immune evasion strategies employed by CMV are the production of viral‐cytokines and chemokine decoy receptors, which disrupt immune cell trafficking and local inflammatory responses (Figure 6). For example, UL111A encodes cmvIL‐10, a viral homolog of human interleukin‐10 (IL‐10), which suppresses proinflammatory cytokine production and impairs antigen‐presenting cell and T‐cell functions [65]. In addition, US28 encodes a viral chemokine receptor homolog that binds multiple host chemokines, thereby disrupting immune cell trafficking and modulating local inflammatory responses [66].

**Figure 6:**
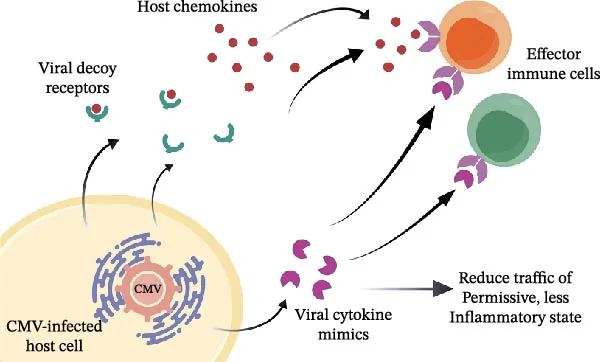
Cytokine and chemokine network manipulation by CMV. CMV encodes viral cytokine mimics, chemokine homologs, and decoy receptors that intercept host cytokine and chemokine signaling. By sequestering host chemokines and altering immune signaling pathways, CMV limits effector immune cell recruitment and dampens local inflammation, thereby promoting a permissive environment for viral persistence and latency. Created with BioGDP.com.

Furthermore, CMV further shapes the inflammatory environment by hijacking cytokine and chemokine signaling pathways [67, 68]. The virus encodes a range of chemokine homologs, chemokine receptor mimics, and soluble cytokine‐binding proteins that distort leukocyte trafficking and dampen antiviral immune activation [69]. Some viral receptors function as “decoys,” sequestering host chemokines to reduce recruitment of effector cells to the sites of infection [57, 70]. Other CMV‐encoded molecules imitate host cytokines and skew immune responses toward more permissive, less inflammatory states [71]. This systemic reprograming of immune communication enables CMV to sustain persistence and latency within immunocompromised hosts. Thus, through these combined mechanisms, CMV reshapes local immune communication to limit effector cell recruitment and sustain a permissive environment for long‐term persistence.

## 5. Mechanisms of Immune Evasion by BKV

The integrated mechanisms by which BKV evades both adaptive and innate immune responses are described as follows.

### 5.1. BKV Infection Alters Antigen Processing and Presentation

BKV persistence and reactivation are closely linked to its ability to reduce immune visibility, particularly to virus‐specific T cells [72]. Antiviral CD8^+^ T cell response against BKV predominantly targets early viral proteins, such as the LTAg [72–74]. LTAg is a key early regulatory protein encoded by the polyomavirus [73, 74]. LTAg is expressed early during infection and localizes predominantly to the nucleus; these features may reduce the availability of viral peptides for MHC class I loading and thereby contribute to suboptimal antigen presentation and reduced CD8^+^ T‐cell‐mediated immune surveillance [75, 76]. This strategy allows infected renal epithelial cells to escape from cytotoxic T lymphocyte‐mediated clearance, particularly under the condition of immunosuppression (Figure 7).

**Figure 7:**
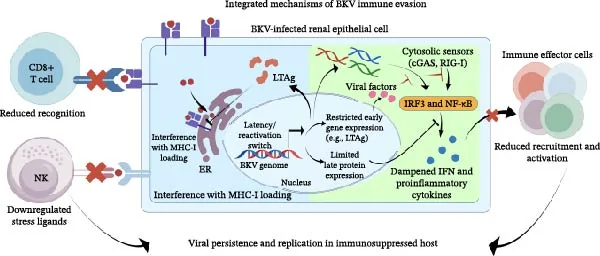
Integrated mechanisms of BK virus immune evasion. BKV persistence and reactivation rely on coordinated modulation of antigen presentation, viral gene expression, and innate immune signaling. Early viral proteins, particularly large LTAg, are the main targets of antiviral CD8^+^ T cell responses; however, restricted expression patterns and intracellular localization of LTAg limit peptide availability for MHC class I loading, reducing T cell recognition. BKV infection is also associated with weak induction of cellular stress responses and limited upregulation of NKG2D ligands, constraining NK cell activation. In parallel, BKV tightly controls viral transcription, maintaining restricted early gene expression and minimal late protein synthesis. Additionally, innate immune sensing via TLR3, RIG‐I and cGAS is attenuated, leading to reduced IRF3 and NF‐κB activation and dampened interferon and cytokine responses. Together, these mechanisms promote immune evasion and viral persistence, particularly in immunosuppressed hosts. Created with BioGDP.com.

Furthermore, BKV infection is also characterized by weak induction of cellular stress responses, resulting in limited upregulation of NKG2D ligands and reduced NK cell activation [77, 78]. This results in reduced immune recognition of BKV‐infected cells.

### 5.2. Viral Gene Expression Control

A central feature of BKV persistence is its ability to modulate the timing and magnitude of viral gene expression [79, 80]. During latency, BK viral transcription is largely silenced, and only a restricted set of viral sequences is expressed, limiting antigen production and immune sensing [28]. When immunosuppression permits viral reactivation, structural and regulatory changes within the NCCR may fine‐tune early gene expression to favor replication while limiting abundant late structural protein expression [1, 28, 81, 82]. This staged expression pattern allows early antigens, including LTAg and small t antigens, to support viral DNA replication before substantial capsid protein production. Late structural proteins such as VP1 are prominent immune targets, particularly for humoral immunity and some T‐cell recognition, whereas LTAg appears to play a more prominent role in cellular immunity, especially BKV‐specific CD8^+^ T‐cell activation [1, 80, 80, 83]. This precise control of transcription enables BKV to switch between silent persistence and productive infection while minimizing immune recognition.

### 5.3. Suppression of Innate Immunity by BKV

BKV also employs multiple strategies to attenuate innate antiviral defenses. Rather than completely failing to trigger host sensing, BKV appears to induce only limited innate immune responses in infected cells, likely reflecting both inefficient recognition of viral nucleic acids and active suppression of downstream signaling [84, 85]. Pattern‐recognition pathways that may be affected include TLR3, RIG‐I‐like receptor signaling, and the cGAS–STING axis, which normally detect foreign nucleic acids and promote type I interferon responses [86, 87], and TLR9 and AIM‐2 like receptor (ALR) may also be involved. The overall effect is a blunted antiviral state that facilitates viral persistence and replication. By dampening the activation of key transcription factors such as IRF3 and NF‐κB, BKV thereby limits the production of type I interferons (Figure 5) and other antiviral mediators capable of restricting early viral spread [88]. In kidney epithelial cells, BKV modulates cytokine and chemokine expressions to create a microenvironment that is less supportive of NK cell recruitment and innate effector activation [89, 90]. These early immune signaling disruptions create a critical replication window for the virus ahead of robust antiviral response induction with heightened consequences in immunosuppressed patients.

## 6. Family‐Level Comparison of Immune Evasion Strategies

While CMV and BKV belong to distinct virus families (Herpesviridae and Polyomaviridae, respectively), comparing each with other members of its own family provides insight into their respective evolutionary advantages in immune evasion.

Within the Herpesviridae family, CMV distinguishes itself from α‐herpesviruses (e.g., HSV‐1) and γ‐herpesviruses (e.g., EBV) through its multilayered and functionally redundant immune‐evasive arsenal. HSV‐1 primarily employs a single mechanism, ICP47‐mediated TAP inhibition, to block MHC‐I antigen presentation, while EBV relies mainly on limited IL‐10 mimicry and LMP‐mediated MHC‐I downregulation [91–93]. In contrast, CMV simultaneously targets MHC‐I processing at multiple distinct steps via US2/US11 (proteasomal degradation), US3 (ER retention), and US6 (TAP inhibition) [51–53, 94]. For NK cell evasion, CMV employs both decoy ligands (UL18 engaging LIR‐1/ILT2) and downregulation of activating ligands (UL16/UL142 targeting the NKG2D pathway) [61, 64]. Additionally, CMV encodes a broad repertoire of chemokine decoy receptors (US28) and cytokine homologs (cmvIL‐10) that reshape the local inflammatory environment [65, 66]. This functional redundancy ensures that even if one evasion pathway is compromised, alternative mechanisms remain operative, enabling CMV to establish latency across a remarkably broad range of tissues.

Within the Polyomaviridae family, BKV adopts a fundamentally different strategy compared with that of JCV and MCPyV. Rather than encoding numerous immunomodulatory proteins, BKV achieves immune evasion through functional minimalism: it restricts viral gene expression, sequesters LTAg in the nucleus to limit peptide–MHC‐I availability, and attenuates innate immune sensing pathways (TLR3, RIG‐I, and cGAS) leading to reduced IRF3 and NF‐κB activation [76, 84–86, 88]. Compared with JCV, which benefits from immune‐privileged sites within the central nervous system or MCPyV, which employs LTAg truncation to evade T‐cell recognition, BKV relies primarily on reducing its own immunological visibility rather than actively suppressing host immune components [95, 96]. This minimalistic “stealth” strategy permits BKV to persist silently in renal epithelial cells and reactivate rapidly when immunosuppression permits, without requiring the extensive genetic toolkit characteristic of larger DNA viruses.

The key differences in immune evasion strategies between CMV and BKV, including their family‐level advantages over related viruses, are summarized in Table 2.

**Table 2:** Comparison of immune evasion proteins, host receptors, diagnostic approaches, and family‐level strategies between CMV and BKV.

Category	CMV	BKV	References
Key immune evasion proteins (mechanism)	MHC‐I: US2/US11 (degradation); US3 (ER retention); US6 (TAP inhibition) NK: UL18 (LIR‐1/ILT2 decoy); UL16/UL142 (NKG2D ligands) Cytokines: UL111A (cmvIL‐10); US28 (decoy receptor)	Ag presentation: LTAg nuclear sequestration NK: weak NKG2D ligand induction Innate: attenuated TLR3/RIG‐I/cGAS → ↓IRF3/NF‐κB Gene control: NCCR‐restricted expression	CMV: [51–53, 59, 62–66] BKV: [75–80, 84–88]
Host receptors/entry factors	PDGFRα (fibroblasts); NRP2 (epithelial/endothelial)	GD1b, GT1b gangliosides (via VP1)	CMV: [17, 37–40] BKV: [42, 43]
Diagnostic methods	qPCR (plasma/whole blood); IGRA, ELISpot, ICS/flow cytometry	qPCR (plasma/urine); IFN‐γ‐based assays (limited standardization)	CMV: [97, 98] BKV: [99–101]
Family‐level advantage	Herpesviridae (vs HSV‐1, EBV): multilayered redundancy—targets MHC‐I at multiple steps (US2/US3/US6/US11); suppresses NK via decoy (UL18) and ligand downregulation (UL16/UL142); manipulates chemokines (US28/cmvIL‐10). Enables broad tissue latency.	Polyomaviridae (vs JCV, MCPyV): functional minimalism (“stealth”)—restricts its own visibility via limited gene expression, LTAg nuclear sequestration, and attenuated innate sensing. Permits silent persistence in renal epithelium.	CMV: [51–53, 61, 64–66, 94] BKV: [84–86, 88, 95, 96]

## 7. Diagnosis and Clinical Management

CMV and BKV pose a major clinical threat to immunocompromised individuals [102–105]. These include organ transplant recipients and patients receiving long‐term immunosuppressive therapy [103, 104]. Viral immune evasion strategies complicate early diagnosis and limit therapeutic efficacy. Effective clinical management therefore requires sensitive diagnostic tools and careful longitudinal monitoring.

### 7.1. Current Molecular and Immunological Diagnostic Tools

The current molecular and immunological approaches used for the diagnosis and monitoring of CMV and BKV infection are shown in Figure 8.

**Figure 8:**
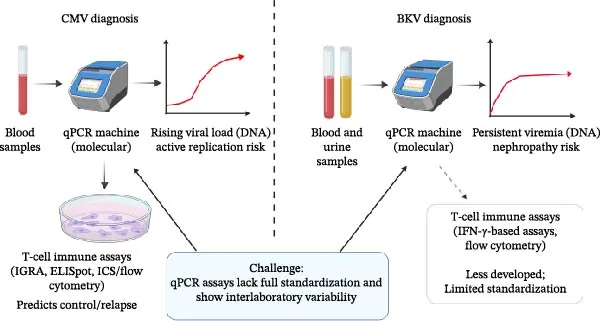
Diagnostic approaches for CMV and BK virus infection. Quantitative polymerase chain reaction (qPCR)‐based viral load monitoring forms the basis of diagnosis and risk assessment for both infections. T‐cell immune assays provide complementary information for CMV, including interferon‐γ release assays (IGRAs), enzyme‐linked immunospot (ELISpot), and intracellular cytokine staining (ICS)/flow cytometry, but remain less developed for BKV, where interferon‐γ (IFN‐γ)‐based assays and flow cytometry have limited standardization. qPCR assays also lack full standardization and show interlaboratory variability. Created with BioGDP.com.

#### 7.1.1. Molecular Diagnostics

Quantitative PCR is the primary method for detecting CMV and BKV infections [97, 99]. The assay can be used to measure viral DNA in blood, urine, or tissue samples. Viral loads are more informative than other qualitative detections [106]. It can guide therapeutic decision in most clinical settings [107].

For CMV infection, the viral DNA levels in plasma or whole blood are routinely monitored [98]. A rising viral load indicates active replication and risk of disease progression [104]. For BKV infection, viral DNAs in urine and plasma are measured to identify early reactivation [100]. Persistent viremia is strongly associated with BKV‐associated nephropathy [5]. Despite their utility, PCR assays lack full standardization [108]. Interlaboratory variability remains a challenge [108], which limits direct comparison across studies and clinical monitoring.

#### 7.1.2. Immunological Diagnostics

Immune‐based assays provide complementary information on the host antiviral capacity.

For CMV, virus‐specific CD4^+^ and CD8^+^ T‐cell responses can be assessed using interferon‐γ release assays (IGRAs), enzyme‐linked immunospot (ELISpot)‐based assays, or intracellular cytokine staining (ICS) by flow cytometry. Stronger CMV‐specific T‐cell responses are generally associated with improved viral control and a lower risk of relapse after treatment, whereas weak or absent responses are associated with recurrent or persistent infection.

For BKV, immune monitoring is less developed. BKV‐specific CD4^+^ and CD8^+^ T‐cell responses have been evaluated mainly using interferon‐γ‐based assays and flow cytometry after peptide stimulation, but these assays are not yet widely standardized [101, 109]. In general, stronger BKV‐specific T‐cell responses are associated with lower viral loads and viral clearance, whereas impaired responses are associated with persistent viremia and BKV‐associated nephropathy [101].

### 7.2. Antiviral Therapies: Efficacy and Limitations

#### 7.2.1. Antiviral Treatment of CMV

Several antiviral drugs are available for CMV infection, mostly targeting viral DNA replication. Conventional anti‐CMV drugs, including ganciclovir, valganciclovir, foscarnet, and cidofovir, effectively reduce viral loads during clinical treatment [104], but they do not eliminate latent infection [110]. Furthermore, CMV frequently reactivates after treatment cessation [111]. This is especially common in patients with impaired T‐cell immunity [112, 113]. Long‐term therapy is further limited by toxicity. Myelosuppression and nephrotoxicity are frequent adverse effects [114–116]. Drug resistance can emerge under prolonged antiviral pressure [117]. Mutations in viral polymerase or terminase genes reduce treatment efficacy [118]. Resistant CMV strains are difficult to manage and often require alternative drugs such as foscarnet, cidofovir, or maribavir [104, 110]. Thus, more effective control measures are still in great demand.

#### 7.2.2. Therapeutic Challenges in BKV Infection

No specific antiviral drug for the control of BKV infection is currently commercially available. Clinical management relies primarily on reducing immunosuppressive therapy [6]. This approach aims to restore antiviral immune control [1]. However, reduction of immunosuppressive drugs poses a significant risk to patients with organ transplantation [105, 119]. Graft rejection is a major concern in organ transplant recipients. Antiviral drugs with off‐label use show inconsistent efficacy [111, 120]. These outcomes reflect limited drug susceptibility and effective immune evasion by BKV.

### 7.3. Challenges in Monitoring and Drug Resistance

A major challenge is distinguishing viral persistence from clinically relevant reactivation. Both CMV and BKV can maintain low‐level replication with minimal antigen expression [1, 121]. This state enables immune evasion and rapid reactivation [121]. As viral loads may fluctuate near diagnostic thresholds [104, 122], such patterns complicate clinical interpretation. A single measurement is often insufficient for decision making [104, 123, 124].

Prolonged antiviral exposure promotes resistance development [118, 125]. For CMV, resistance mutations directly impair treatment success [118], while for BKV, regulatory genome alterations may enhance replication efficiency [81]. These changes do not necessarily increase immunogenicity. In addition, these challenges highlight the need for integrated monitoring strategies [81, 126]. Viral load data should be combined with immune function assessment [127]. Such approaches may improve risk assessment and guide individualized clinical management [128].

The major immunocompromised patient populations at risk for CMV and BKV reactivation, including those with concurrent infections, are summarized in Table 3.

**Table 3:** Immunocompromised patient populations at risk for CMV and/or BKV reactivation.

Patient population	CMV	BKV	Coinfection	Major clinical consequences	References
Solid organ transplant recipients	✓✓	✓✓	✓✓	CMV: pneumonitis, GI injury, retinitis; BKV: nephropathy	[103, 104]
Hematopoietic stem cell transplant recipients	✓✓	✓✓	✓	CMV: pneumonitis; BKV: hemorrhagic cystitis	[102–105]
Patients undergoing chemotherapy	✓✓	—	—	CMV reactivation, systemic inflammation	[102–105]
Advanced HIV/AIDS	✓✓	—	—	CMV: retinitis, pneumonia, and GI injury	[102–105]

*Note:* ✓✓ = high risk; ✓ = at risk. Coinfection is particularly common in solid organ and hematopoietic stem cell transplant recipients.

A comprehensive comparison of CMV and BKV across cellular tropism, entry mechanisms, immune evasion strategies, diagnostic approaches, and therapeutic options is provided in Table 2.

## 8. Emerging Therapeutic Strategies

Emerging therapeutic strategies for CMV and BKV infection increasingly emphasize immune restoration, alternative antiviral targeting, and precision‐guided clinical decision making. As shown in Figure 9, the flowchart provides a conceptual overview of these complementary approaches and highlights distinct therapeutic trajectories for CMV and BKV infections in immunocompromised patients.

**Figure 9:**
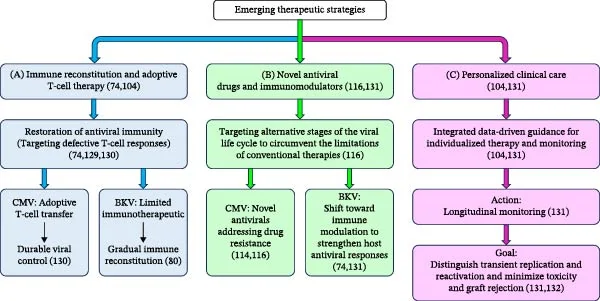
Conceptual framework of emerging therapeutic strategies for CMV and BKV infections. The diagram illustrates three integrated approaches. (A) Immune‐based therapy, emphasizing virus‐specific T‐cell transfer for CMV and immune reconstitution for BKV. (B) Pharmacological intervention, featuring novel antivirals for resistant CMV and immunomodulators for BKV. (C) Personalized clinical care, utilizing longitudinal immune‐monitoring to distinguish viral replication from clinical reactivation. The overarching goal is to achieve durable viral control while minimizing adverse effects or toxicity. This is an original figure created by the authors. References supporting the individual statements are provided within the figure and in the References section.

### 8.1. Immune Reconstitution and Adoptive T‐Cell Therapy

Restoration of antiviral immunity is increasingly recognized as a key strategy for controlling CMV and BKV infections in immunocompromised patients [74, 104]. Persistent infection is largely driven by defective virus‐specific T‐cell responses rather than uncontrolled viral replication alone [47, 129, 130]. For the control of CMV infection, adoptive transfer of CMV‐specific CD4^+^ and CD8^+^ T cells has demonstrated durable viral control in selected high‐risk patients [130]. In contrast, immune‐based interventions for BKV control remain limited due to low antigen expression, although gradual immune reconstitution may still contribute to improved viral containment [80].

### 8.2. Novel Antiviral Drugs and Immunomodulators

New antiviral agents, such as letermovir and maribavir, aim to overcome the limitations of conventional therapies by targeting alternative stages of the viral life cycle [116]. For CMV, these drugs expand options for resistant infections and improve tolerability [110, 116]. However, they remain suppressive and do not eliminate viral latency [110]. For BKV, the absence of approved antivirals has shifted attention toward immunomodulatory approaches that enhance the host antiviral capacity rather than directly inhibiting viral replication [74, 131].

### 8.3. Personalized Clinical Care in Immunocompromised Patients

The clinical course of CMV and BKV infections varies widely among immunocompromised individuals. Personalized clinical care integrate viral load dynamics, immune status, and patient‐specific risk factors to guide therapeutic decisions [104, 131]. Longitudinal monitoring allows for discrimination between transient viral replication and clinically significant reactivation [131]. Such immune‐guided strategies may improve viral control effectiveness while minimizing treatment‐related toxicity and graft rejection [131, 132].

## 9. Summary and Future Perspectives

CMV and BKV infections pose a severe threat to individuals with compromised immunity, in particular, to organ transplant patients with immunosuppression due to routine medication. A complete understanding of the mechanisms by which CMV and BKV evade host immune responses and establish persistent infection will be of great value and guidance to the development of effective therapeutic strategies. CMV and BKV illustrate how structurally and genetically distinct viruses converge on common principles of immune evasion to achieve persistent infection. Reframing viral latency as a spectrum of immune‐adapted states rather than a binary condition provides a conceptual framework for the development of next‐generation diagnostics and immune‐centered therapeutic strategies for the control of CMV and BKV infections in immunocompromised host.

## Funding

This work was supported by grants from the Earmarked Fund for CARS‐40, China.

## Disclosure

The funders had no role in the design of the study; in the collection, analyses, or interpretation of data; in the writing of the manuscript; or in the decision to publish the results.

## Conflicts of Interest

The authors declare no conflicts of interest.

## Data Availability

Data sharing is not applicable to this article as no datasets were generated or analyzed during the current study.
